# Seminal F_2_-IsoP and RvD1 Levels in Idiopathic Infertile Men

**DOI:** 10.3390/biology14040450

**Published:** 2025-04-21

**Authors:** Elena Moretti, Giulia Collodel, Caterina Marcucci, Laura Liguori, Laura Gambera, Cinzia Signorini

**Affiliations:** 1 Department of Molecular and Developmental Medicine, University of Siena, 53100 Siena, Italy; elena.moretti@unisi.it (E.M.); caterin.marcucci@student.unisi.it (C.M.); laura.liguori@student.unisi.it (L.L.); cinzia.signorini@unisi.it (C.S.); 2 Fertility Center, AGI Medica, 53100 Siena, Italy; lauragambera@agimedica.it

**Keywords:** F_2_-isoprostanes, human semen, idiopathic infertility, oxidative stress, resolvin D1

## Abstract

30 percent of infertile men are diagnosed with idiopathic infertility. This study aimed to assess oxidative stress in the semen of 77 patients with idiopathic infertility by measuring F_2_-isoprostane (F_2_-IsoP), resolvin D1 (RvD1) levels, and semen parameters. The presence and localization of 8-IsoProstaglandin F_2α_ were determined using immunofluorescence. No significant correlations were observed for F_2_-IsoP and RvD1 levels with the semen variables. Based on F_2_-IsoP levels, individuals were classified into two groups: Group 1 (F_2_-IsoPs ≤ 29.96 ng/mL, 51%) and Group 2 (F_2_-IsoPs > 29.96 ng/mL, 49%). In comparison to Group 1, Group 2 showed significantly higher F_2_-IsoP levels (13.33 ng/mL vs. 44.80 ng/mL; *p* < 0.05), a lower progressive motility percentage (30% vs. 25%; *p* < 0.05), and increased RvD1 levels (36.09% vs. 44.94%). Immunofluorescence analysis revealed a different localization of 8-IsoProstaglandin F_2α_ in the ejaculated sperm of Group 1 compared to that observed in Group 2. A weak signal was detected in the sperm tail (Group 1, 79.1% vs. Group 2, 36.9; *p* < 0.01). In spermatozoa of Group 2 patients, a strong signal in the acrosome, midpiece, and tail was highlighted. These findings suggest the need to test oxidative stress during routine semen analysis in patients with idiopathic infertility to improve diagnosis and treatment.

## Simple Summary

A significant percentage of male infertility is classified as idiopathic infertility, defined as abnormality in at least one semen parameter, with no previous history of diseases affecting fertility. In this paper, we investigated whether the presence of oxidative stress might be involved in idiopathic infertility. Oxidative stress is strongly associated with inflammatory processes, creating a self-reinforcing cycle that impairs sperm parameters and, consequently, male fertility. In the context of oxidative stress and inflammation, F_2_-Isoprostane (F_2_-IsoP), a prostaglandin-like compound produced from non-enzymatic peroxidation of arachidonic acid, and resolvin D1 (RvD1), a resolvin that functions as a lipid mediator in the resolution of inflammation, were assessed in semen. The main results obtained in this study indicate reduced progressive sperm motility in patients defined as idiopathic infertile with higher levels of F_2_-IsoPs and RvD1. For these reasons, more advanced tests are needed to accurately diagnose male infertility by evaluating the environment provided by the seminal plasma. In particular, in idiopathic infertile patients with reduced motility, it is likely that infertility may be associated with OS, which is worth investigating.

## 1. Introduction

The World Health Organization (WHO) estimates that 9% of couples worldwide face fertility issues, with 50% of cases attributed to males [1]. Despite advances in the understanding of male infertility, it has been estimated that 30% of infertility is associated with idiopathic causes [1,2]. This condition of idiopathic male infertility is defined as abnormality in at least one semen parameter, with no previous history of diseases affecting fertility, and normal findings on physical examination and genetic and laboratory testing [3]. Therefore, other examinations are necessary to provide information that remains unrevealed by currently used semen analysis methods, as none of the seminal parameters detectable through these analyses test the real fertilizing capacity of the sperm; thus, semen analysis is never a prognostic fertility tool [2,4]. The study of genes involved in pivotal steps of spermatogenesis, such as germ cell proliferation, meiosis, and spermiogenesis, may increase the diagnosis of idiopathic patients with an abnormal sperm count, motility, and/or morphology [5,6]. Global sperm methylation or methylation of imprinted genes involved in human sperm quality might also represent good candidates for investigation in idiopathically infertile patients [7].

The significant role of oxidative stress (OS) in the pathogenesis of male infertility has been supported by several authors in recent years. Agarwal and colleagues [8] suggested the term and concept of “Male Oxidative Stress Infertility (MOSI)” and found that many patients previously categorized as having idiopathic male infertility actually have MOSI. Nevertheless, a considerable proportion of male infertility cases continues to be classified as idiopathic or unexplained, underscoring the critical need for advanced research to elucidate the underlying mechanisms [9].

Therefore, idiopathic infertility is a heterogeneous condition. Some groups have not found an increase in OS in patients with idiopathic infertility [10,11], while others have associated idiopathic infertility with a pathological oxidative condition [12,13].

These data might be explained by uncovering the real meaning of idiopathic infertility, as it is challenging to categorize patients with similar traits. Certainly, in the semen of many males with idiopathic or unexplained infertility, OS has been identified; yet, other genetic, epigenetic, or unidentified environmental factors could also play a role [14].

OS is strongly associated with inflammatory processes, creating a self-reinforcing cycle that impairs sperm parameters and, consequently, male fertility. In this context, reactive oxygen species (ROS) play a dual role: they regulate the progression of inflammation and, when they exceed the endogenous antioxidant capacity, they compromise sperm’s fertilization potential [15]. F_2_-Isoprostanes (F_2_-IsoPs), structural isomers of prostaglandin F_2α_ (PGF_2α_), are formed via free-radical-mediated peroxidation of polyunsaturated fatty acids (PUFAs) esterified in membrane phospholipids. These compounds have been established as powerful markers of in vivo lipid peroxidation (LPO), demonstrating their potential to accurately reflect the OS status in various human diseases [16], including male infertility [17]. Furthermore, Resolvin D1 (RvD1), derived from docosahexaenoic acid, belongs to the class of specialized pro-resolving mediators (SPMs), and it is linked to inflammatory male infertility and reduced sperm quality. Elevated RvD1 levels in semen correlate with increased F_2_-IsoPs, sperm necrosis, and reduced motility, particularly in the presence of leukocytospermia and varicocele [18]. Hence, RvD1 emerges as a promising biomarker to be integrated into a set of seminal inflammatory markers, facilitating more accurate diagnosis of inflammatory male infertility and guiding personalized therapeutic approaches.

This study aims to evaluate semen samples from 77 patients with idiopathic infertility by assessing sperm parameters and seminal F_2_-IsoP and RvD1 levels to identify potential biomarkers for this specific form of male infertility. In particular, the possible inclusion of OS markers in routine semen analysis to improve infertility diagnosis and treatment are discussed.

## 2. Materials and Methods

### 2.1. Patients

Semen samples for this investigation were procured from 77 Italian men (age range: 29–39 years) experiencing infertility, who visited the AGI Medica Fertility Center (Siena, Italy) for semen analysis. All participants had a documented history of inability to achieve conception following a minimum of two years of regular, unprotected intercourse. Female factor infertility was excluded prior to participant enrollment. 

Participants meeting the specified inclusion criteria were classified as having idiopathic male infertility, a condition characterized by impaired semen parameters in the absence of identifiable etiological factors. Inclusion criteria stipulated that participants must be non-azoospermic, possess a normal karyotype, exhibit a body mass index below 25 kg/m^2^, and present with serum hormonal profiles within the normal physiological range. Exclusion criteria encompassed the presence of chronic systemic diseases, current or recent treatment with radiotherapy or chemotherapy, diagnosed varicocele, leukocytospermia, or active urogenital infections. Furthermore, lifestyle factors were considered: individuals currently utilizing oral antioxidant supplements (within the preceding four months), those with significant tobacco consumption (defined as >10 cigarettes per day), or reporting a history of recreational drug use or excessive alcohol consumption were excluded from participation

Enrollment was contingent upon participants providing consent for the potential utilization of residual semen sample volume, not required for diagnostic analysis, for scientific research purposes. This study protocol received approval from the institutional ethics committee (Authorization for Clinical Trials pursuant to Ministerial Decree, 19 March 1998; Reference N.1327, 13 December 2018). Written informed consent for participation in the research protocol was obtained from all enrolled individuals prior to sample collection.

### 2.2. Semen Analysis

Semen samples were collected via masturbation into sterile containers following a period of sexual abstinence of 3–5 days. Standard semen parameters were evaluated according to the WHO guidelines [5]. Specifically, sperm motility was assessed and categorized as rapid progressive, slow progressive, non-progressive, or immotile. Sperm morphology was evaluated using pre-stained Testsimplets® slides (Origio, Firenze, Italy). Sperm vitality was determined using the eosin staining technique, employing 0.5% Eosin Y (Thermo Fisher Scientific, Waltham, MA, USA) in a 0.9% aqueous sodium chloride solution. The established lower reference limit for sperm vitality is ≥54%. Following routine semen analysis, samples were aliquoted for subsequent assays. For immunolocalization studies, 100 μL aliquots were washed with phosphate-buffered saline (PBS) and subsequently smeared onto microscope slides for F_2_-IsoPs analysis. Following routine semen analysis, samples were aliquoted for subsequent assays. 

### 2.3. F_2_-Isoprostanes (F_2_-IsoPs) Determination

Total F_2_-IsoP concentrations in seminal plasma were quantified following established methodologies. Initially, samples underwent basic hydrolysis. Subsequently, acidified water and an internal standard, deuterated prostaglandin F_2α_ (PGF_2α−d4_), were added to each sample. Purification was performed using sequential solid-phase extraction. Samples were first loaded onto an octadecylsilane (C18) cartridge (WAT043395, Sep-Pak® Vac C18, 500 mg, Waters, Milford, MA, USA). The eluate from the C18 cartridge was then applied to an aminopropyl (NH_2_) cartridge (WAT054560, Sep-Pak® Vac NH_2_, 500 mg, Waters, Milford, MA, USA).

Following purification, chemical derivatization was performed. The carboxylic acid group of F_2_-IsoPs was converted to its pentafluorobenzyl ester derivative, while the hydroxyl groups were converted to trimethylsilyl ethers. Final quantification was achieved using gas chromatography coupled with negative ion chemical ionization tandem mass spectrometry (GC/NICI-MS/MS) utilizing a TRACE GC and PolarisQ Ion Trap system (Thermo Finnigan, San Jose, CA, USA). F_2_-IsoPs were quantified by monitoring the specific precursor-to-product ion transition for 8−iso−PGF_2α_ (Cayman Chemical, Ann Arbor, MI, USA), the most abundant F_2_-IsoP isomer [19], specifically targeting the fragment ion at *m*/*z* 299. Results were expressed as ng/mL.

### 2.4. Resolvin D1 (RvD1) Determination

Concentrations of Resolvin D1 (RvD1) in seminal plasma were determined using a commercial double-antibody sandwich Enzyme-Linked Immunosorbent Assay (ELISA) kit (MyBioSource, San Diego, CA, USA; Catalog number inferred or add if known) [18]. This assay employed microtiter plate wells pre-coated with an anti-RvD1 monoclonal capture antibody and utilized a biotin-conjugated polyclonal antibody for detection.

The assay was performed according to the manufacturer’s protocol, with the following key steps: standard solutions (containing known RvD1 concentrations) and seminal plasma samples were added to the appropriate wells. The plate was incubated for 90 min.

Wells were washed twice with PBS. The biotinylated detection antibody solution was added to each well, followed by a 60-min incubation. Wells were washed three times with PBS. An avidin-peroxidase enzyme conjugate was added, followed by a 30-min incubation. Wells were washed five times with PBS. The substrate solution, 3,3′,5,5′-tetramethylbenzidine (TMB), was added to initiate the colorimetric reaction. Following color development, the reaction was terminated by the addition of a stop solution (typically within 30 min of substrate addition).

The optical density (absorbance) of each well was measured spectrophotometrically at a wavelength of 450 nm using a compatible microplate reader.

All standards and samples were assayed in duplicate. A standard curve was generated using calibrators provided in the kit at the following concentrations: 2000, 1000, 500, 250, 125, 62.5, 31.2, and 0 pg/mL. RvD1 concentrations in the samples were interpolated from this standard curve and expressed in pg/mL.

### 2.5. Immunolocalization of F_2_-IsoPs

Immunofluorescence analysis was performed in 10 patients of Group 1 and 10 patients of Group 2 to evaluate the localization of F_2_-IsoPs [19]. First, 100 μL of each sample were washed in PBS, smeared on glass slides, and air-dried. The smeared slides were fixed in methanol at −20 °C for 20 min, followed by acetone at −20 °C for 5 min, and rehydrated in PBS for 10 min at room temperature before the reaction. Then, the slides were treated with PBS-bovine serum albumin (BSA) 1% and 5% of normal goat serum (NGS) for 20 min and incubated overnight at 4 °C with a rabbit polyclonal anti-8-iso-PGF_2α_ antibody (Abcam, Cambridge, UK), diluted 1:100 in PBS-BSA 0.1% and NGS 1%. The reaction was detected using a goat anti-Rabbit IgG Secondary Antibody Alexa Fluor™ 488 (Thermo Fisher Scientific, Waltam, MA, USA) diluted 1:500 in PBS-BSA 0.1% and NGS 1%. Specificity of binding was confirmed by the negative staining using the diluent (PBS-BSA 0.1% and NGS 1%) and omitting the primary antibody. Sperm nuclei were stained with 4,6-diamidino-2-phenylindole (DAPI) solution (Vysis, Downers Grove, IL, USA) diluted 1:20,000 in PBS for 10 min at room temperature. Finally, the slides were rinsed in PBS and mounted with 1,4-diazabicyclo [2.2.2] octane (Sigma-Aldrich, St. Louis, MO, USA). The slides were observed and evaluated with a Leica DMI 6000 Fluorescence Microscope (Leica Microsystems, Wetzlar, Germany), and the images were acquired by the Leica AF6500 Integrated System for Imaging and Analysis (Leica Microsystems, Wetzlar, Germany).

### 2.6. Statistical Analysis

Statistical analyses were conducted using the SPSS software package, version 17.0 for Windows (SPSS Inc., Chicago, IL, USA). The Kolmogorov–Smirnov test was employed to assess the normality of variable distributions. Spearman’s rank correlation coefficient (ρ) was utilized to evaluate the associations between the variables under investigation. The Mann–Whitney U-test was applied to compare differences between the two groups. Data are presented as median values with interquartile ranges (25th–75th percentiles). A *p*-value of less than 0.05 was considered indicative of statistical significance.

## 3. Results

Semen samples were collected from 77 patients with idiopathic infertility who met the inclusion criteria outlined in the Materials and Methods section. The median values and interquartile ranges (25th–75th percentiles) of the variables assessed in the overall study population are presented in Table 1. Reference values corresponding to the 5th percentile were provided for semen parameters, as well as for normal levels of F_2_-IsoPs and RvD1.

To investigate potential correlations among the variables studied, Spearman’s rank correlation coefficient was applied to the entire study population. Correlations were assessed across the full cohort to identify trends indicating whether changes in one variable were associated with changes in another. No significant correlations were observed between F_2_-IsoPs or RvD1 and any of the semen parameters evaluated. However, sperm concentration, progressive motility, normal morphology, and vitality were found to be positively correlated, as shown in Table 2.

Successively, patients were divided into two groups according to the levels of F_2_-IsoPs (Group 1 ≤ 29.96 ng/mL and Group 2 > 29.96 ng/mL, as reported in the study of Moretti and colleagues [20]). Group 1 was composed of 39 patients and Group 2 of 38 patients, representing, respectively, 51% and 49% of the examined population.

Spearman’s rank correlation coefficient was used to analyze the relationships among all studied variables within Group 1 and Group 2. No significant correlations were found in either group between semen parameters and levels of F_2_-IsoPs or RvD1. Subsequently, the variables were compared between the two groups, as presented in Table 3.

**Figure 1:**
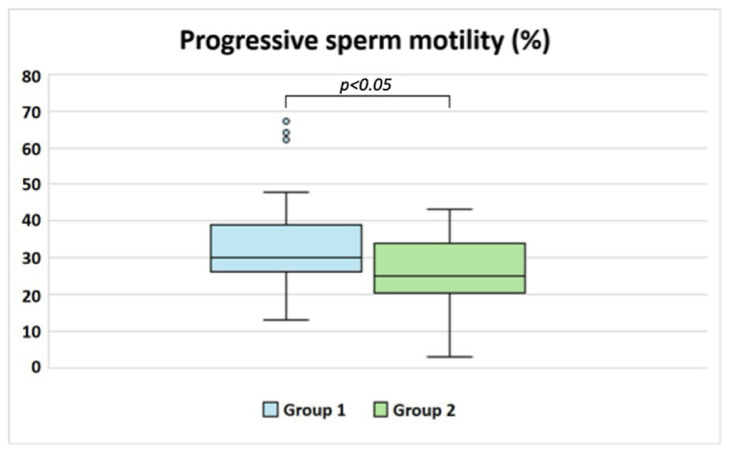
Box plot of the percentage of progressive sperm motility evaluated in idiopathic patients grouped according to their F_2_-IsoP level (cut-off 29.96 ng/mL [20]), a marker of LPO.

**Figure 2:**
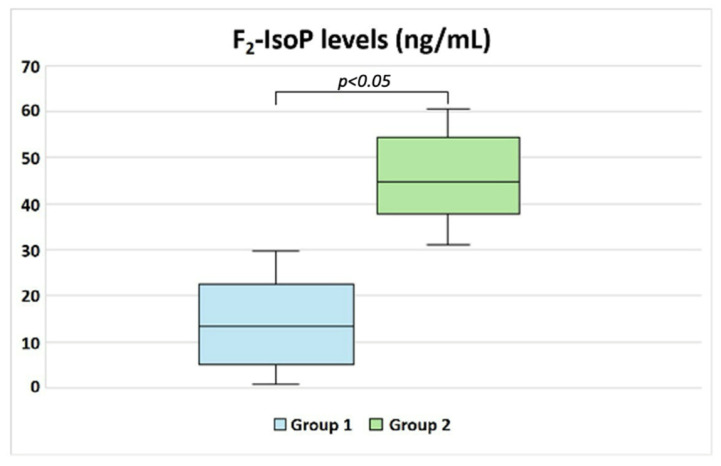
Box plot showing the F_2_-IsoP levels measured in the seminal plasma of idiopathic patients grouped according to their F_2_-IsoP level (cut-off 29.96 ng/mL [20]). The difference between the two groups was significant.

Patients in Group 1 exhibited significantly higher progressive sperm motility (*p* < 0.05; Table 3, Figure 1), along with a slight increase in the percentages of morphologically normal sperm and sperm vitality. Additionally, Group 1 showed lower levels of RvD1 compared to Group 2 (Table 3). A significant difference was also observed in F_2_-IsoP levels between the two groups (*p* < 0.05; Table 3, Figure 2), with Group 2 displaying higher concentrations, as expected.

**Figure 3:**
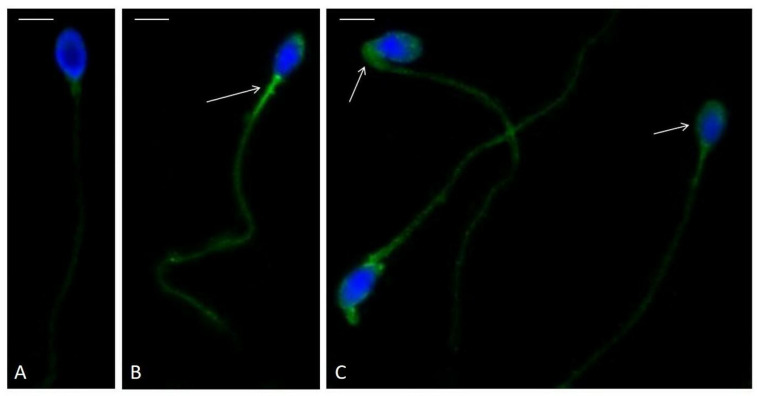
Immunofluorescence staining of spermatozoa using a polyclonal antibody against 8-iso-PGF_2_α. (**A**) Spermatozoa from a patient in Group 1 exhibit weak labeling in the flagellum. (**B**,**C**) Spermatozoa from patients in Group 2 show more intense 8-iso-PGF_2_α staining localized to the mitochondrial sheath ((**B**), arrows), as well as the acrosomal region and cytoplasmic residues ((**C**), arrows). Nuclei were counterstained with 4′,6-diamidino-2-phenylindole (DAPI). Scale bars (**A**–**C**): 5 µm.

Subsequently, spermatozoa from idiopathic infertility patients, categorized into Group 1 and Group 2, were analyzed by immunofluorescence to localize F_2_-IsoPs (8-iso-PGF_2_α). In Group 1, 79.1% of spermatozoa (Figure 3A) exhibited a faint signal localized to the tail. In contrast, only 36.9% of spermatozoa from Group 2 showed this pattern, a difference that was statistically significant (*p* < 0.01). The majority of spermatozoa in Group 2 displayed intense immunolabeling in the acrosomal region, tail, and cytoplasmic residues (Figure 3B,C).

## 4. Discussion

In various pathologies associated with male infertility [21,22,23], including some cases of idiopathic infertility [3,24], OS is a common contributing factor. Recently, a new category of male infertility, termed “MOSI”, was proposed to include idiopathic infertile men with OS [24,25]. Despite these advancements, OS assessment is not yet a standard component of the evaluation process for infertile men, and remains a challenging condition to diagnose and treat [26]. Aitken has emphasized the urgent need for a diagnostic test for OS to aid in male infertility diagnosis and guide treatment decisions. It is crucial to reserve antioxidant therapy for infertile patients who exhibit signs of OS, avoiding indiscriminate administration in the absence of a significant OS contribution to infertility [27].

One indirect method for assessing OS damage is the evaluation of LPO. Malondialdehyde, a major product of LPO, has traditionally been used to gauge oxidative damage [21]. However, F_2_-IsoPs, byproducts of non-enzymatic oxidation of arachidonic acid, have recently been identified as ideal biomarkers due to their chemical stability both in vitro and in vivo and their measurable presence through non-invasive techniques [17,28]. The last couple of decades has shown a growing interest in the role of isoprostanes in male-infertility-related disorders; they seem to be relevant in the evaluation of OS in seminal plasma and spermatozoa [13,17,29,30,31].

Generally, OS is linked to inflammatory processes, and male infertility can result from inflammation-mediated OS caused by factors such as varicocele, smoking, obesity, leukocytospermia, urogenital infections [21,30], and idiopathic infertility [8]. Consequently, the identification of reliable OS and inflammation markers in semen remains a crucial area of research, particularly in cases where a precise diagnosis is lacking, such as in idiopathic infertility.

Building on these premises, the current study aims to contribute to the understanding of OS and inflammation in a cohort of 77 idiopathic infertile patients by measuring seminal levels of F_2_-IsoPs as an OS marker and RvD1 as an inflammation marker. RvD1, a member of the specialized SPMs class, plays a key role in inflammation resolution pathways. Dysregulation of SPMs has been implicated in various pathological conditions, including male infertility, where inflammation adversely affects spermatogenesis [30].

The entire study cohort exhibited reduced motility compared to the WHO 2021 guidelines (5th percentile) [5], a slight increase in RvD1 levels, and F_2_-IsoP levels similar to those reported in the semen of fertile men in previous research [19,20]. These findings, coupled with the lack of correlations between F_2_-IsoPs, RvD1, and semen parameters—observed in other pathological inflammatory male reproductive conditions [18]—suggest that OS may not be involved in the overall cohort of idiopathic infertile men in this study. However, in 2022, our research group established a cut-off value for F_2_-IsoP levels in seminal plasma that discriminates fertile men (≤29.96 ng/mL) with low LPO levels from infertile patients with high LPO levels [20]. This cut-off was used to categorize the idiopathic patients in the current study into Group 1 (low LPO) and Group 2 (high LPO). Approximately half of the idiopathic patients were assigned to Group 2, where they exhibited increased seminal plasma levels of F_2_-IsoPs, decreased progressive sperm motility, and a non-significant increase in RvD1. Additionally, immunofluorescence analysis revealed that spermatozoa from patients in Group 2 had elevated concentrations of F_2_-IsoPs in the tail, acrosome, and cytoplasmic residue. This observation aligns with the fact that isoprostanes are generated in situ in membrane phospholipids by ROS and subsequently released into seminal plasma by phospholipase A_2_ (PLA_2_) activity [32]. The absence of immunofluorescent staining in spermatozoa from idiopathic infertile men in Group 1 (with normal seminal F_2_-IsoP levels) confirms the low LPO levels, as previously reported in spermatozoa of fertile men [33]. These findings suggest that oxidative lipid damage may contribute to the reduction in progressive motility observed in spermatozoa from Group 2. However, while F_2_-IsoP levels in Group 2 were elevated, they were not as high as those seen in infertile men with other conditions, such as varicocele [20].

This study concurs with prior investigations [18] that report a less pronounced oxidative metabolism of fatty acids in patients with idiopathic infertility compared to those with conditions like varicocele or leukocytospermia, where pro-inflammatory cytokines regulate both pro- and antioxidant activities in the male reproductive tract [34]. A limitation of the current study is the absence of cytokine measurements (e.g., IL-6, IL-8), which would have provided valuable insight into the inflammatory state. The relatively modest increase in RvD1 levels in Group 2, which did not reach statistical significance, may reflect an under-stimulation of the oxidative metabolism of fatty acids in these patients. Increased serum RvD1 levels have been associated with attenuated inflammation and improved disease outcomes in other clinical contexts [35]. If RvD1 production signifies inflammation resolution, the stable levels of RvD1 in idiopathic patients with elevated LPO suggest two possible hypotheses: either inflammation is not a key mechanism in the pathophysiology of idiopathic infertility, or there is a diminished ability to resolve inflammation in these patients. Thus, an unresolved subclinical inflammatory process may contribute to oxidative damage. These hypotheses remain open, as inflammatory markers were not evaluated in this study.

While the study population is relatively small, the findings suggest that concomitant increases in F_2_-IsoPs, stable RvD1 levels, and decreased progressive sperm motility may serve as useful markers for categorizing idiopathic infertility. Furthermore, lifestyle factors such as antioxidant supplementation, tobacco use (>10 cigarettes/day), and a history of drug and alcohol consumption were considered in the selected patient group. However, genetic background, living environment, and dietary habits were not controlled for, which may limit the generalizability of the findings in humans.

Another point for discussion concerns the mass spectrometry method used to quantify seminal plasma F_2_-IsoP levels. While mass spectrometry is a sophisticated and sensitive technique for studying OS in male idiopathic infertility, it is time-consuming and not yet suitable for routine laboratory use. Nonetheless, this technique enabled the quantification of lipid oxidative damage in nearly half of the idiopathic patients, confirming that a substantial proportion of idiopathic infertility cases involve LPO due to redox imbalance, which can impair sperm motility. More accessible techniques, such as ELISA, are commercially available, and new tools for assessing both oxidative and antioxidant capacity in seminal plasma are likely to be developed soon. In this regard, a rapid test for diagnosing OS and antioxidant imbalance has been proposed, potentially allowing for antioxidant supplementation as a treatment strategy [36]. Recent developments also suggest that molecules with anti-inflammatory and antioxidant properties could serve as treatments for reproductive diseases (MOXI: male, antioxidants, and infertility) [24].

In conclusion, while standard semen analysis remains the initial diagnostic tool for male infertility, it does not provide insight into the underlying cause or predict fertilization outcomes. Given these limitations, additional reliable tests evaluating the seminal plasma environment are essential for diagnosing male infertility [37,38].

## 5. Conclusions

This study demonstrated that approximately 50% of the patients with idiopathic infertility exhibited elevated seminal levels of F_2_-IsoPs, a recognized marker of OS, along with reduced sperm motility, compared to idiopathic patients with lower F_2_-IsoP levels. These findings suggest that, in idiopathic infertile men with impaired motility, infertility may be associated with OS, warranting further investigation into its potential role in the pathophysiology and management of such cases.

## Figures and Tables

**Table 1:** Median (25th–75th centile) of the considered variables in the group of 77 cases included in this study. F_2_-Isoprostanes (F_2_-IsoPs, ng/mL), Resolvin D1 (RvD1, pg/mL).

Semen Parameters	Median (25th–75th Percentile)	5th Percentile Values [5]
Volume (mL)	3.60 (3.00–4.35)	1.40
Sperm concentration (10^6^× mL)	16.50 (2.03–36.25)	16.00
Sperm progressive motility (%)	28.00 (22.00–35.00)	30.00
Sperm normal morphology (%)	6.00 (4.00–9.00)	4.00
Sperm vitality (%)	70.00 (64.00–75.00)	54.00
Seminal levels	Median (25th–75th percentile)	Control values
F_2_-IsoPs (ng/mL)	29.80 (13.33–44.70)	29.96 [20]
RvD1 (pg/mL)	42.51 (31.88–54.65)	31.20 [18]

**Table 2:** Correlations (Spearman’s coefficient) between the variables considered in 77 individuals. F_2_-Isoprostanes (F_2_-IsoPs, ng/mL), Resolvin D1 (RvD1, pg/mL); * *p* < 0.05.

	Sperm Concentration (10^6^× mL)	Sperm Progressive Motility (%)	Sperm Normal Morphology (%)	Sperm Vitality (%)	F_2_-IsoPs (ng/mL)	RvD1 (pg/mL)
Sperm concentration (10^6^× mL)	1					
Sperm progressive motility (%)	r = 0.34 *	1				
Sperm normal morphology (%)	r = 0.38 *	r = 0.30 *	1			
Sperm vitality (%)	r = 0.35 *	r = 0.43 *	r = 0.49 *	1		
F_2_-IsoPs (ng/mL)	r = 0.14	r = −0.18	r = −0.06	r = −0.11	1	
RvD1 (pg/mL)	r = −0.01	r = −0.21	r = 0.25	r = −0.05	r = 0.13	1

**Table 3:** Median (25th–75th centile) of seminal characteristics, seminal F_2_-isoprostanes (F_2_-IsoPs, ng/mL) and Resolvin D1 (RvD1, pg/mL) assayed in semen samples of 77 men divided into two groups according to their F_2_-IsoP level [20]. Statistics are also reported, ns, not significant.

Semen Parameters	Median (25th–75th Percentile)		Statistics
	Group 1	Group 2	
Volume (mL)	3.50 (3.00–4.50)	3.80 (3.00–4.00)	ns
Sperm concentration (10^6^× mL)	9.55 (1.55–29.50)	18.00 (7.25–47.50)	ns
Sperm progressive motility (%)	30.00 (26.50–38.50)	25.00 (21.00–33.75)	*p* < 0.05
Sperm normal morphology (%)	6.00 (4.00–9.00)	5.00 (4.25–8.00)	ns
Sperm vitality (%)	72.00 (65.00–78.00)	69.50 (59.25–75.00)	ns
Seminal levels			
F_2_-IsoPs (ng/mL)	13.33 (5.30–22.11)	44.80 (37.87–53.94)	*p* < 0.05
RvD1 (pg/mL)	36.09 (31.85–46.47)	44.94 (34.00–56.48)	ns

## Data Availability

The data generated and analyzed during this study are included in this published article and are available from the corresponding author. The data are not publicly available due to the privacy of the patients.

## Abbreviations

OS	Oxidative stress
F_2_-IsoP	F_2_-Isoprostane
RvD1	Resolvin D1
WHO	World Health Organization
MOSI	Male oxidative stress infertility
ROS	Reactive oxygen species
PGF_2α_	Prostaglandin F_2α_
PUFAs	Polyunsaturated fatty acids
LPO	Lipid peroxidation
SPMs	Specialized pro-resolvin mediators
PBS	Phosphate-buffered saline
GC/NICI-MS/MS	Gas chromatography/negative ion chemical ionization tandem mass spectrometry
BSA	Bovine serum albumin
NGS	Normal goat serum
DAPI	4,6-diamidino-2-phenylindole
